# Heregulin, a new interactor of the telosome/shelterin complex in human telomeres

**DOI:** 10.18632/oncotarget.4962

**Published:** 2015-07-22

**Authors:** Javier A. Menendez, Louisa Benboudjema, Luciano Vellon, Miguel A. Rubio, Ingrid Espinoza, Judith Campisi, Ruth Lupu

**Affiliations:** ^1^ ProCURE (Program Against Cancer Therapeutic Resistance), Metabolism & Cancer Group, Catalan Institute of Oncology (ICO), Girona, Spain; ^2^ Girona Biomedical Research Institute (IDIBGI), Girona, Spain; ^3^ Evanston Northwestern Healthcare Research Institute, Evanston, IL, USA; ^4^ IBYME, CONICET-Laboratorio de Immunohematología, Buenos Aires, Argentina; ^5^ Laboratory of Hematology Service, Institut d'Investigació Biomèdica Sant Pau, Hospital de la Santa Creu i Sant Pau, Barcelona, Spain; ^6^ Department of Biochemistry, University of Mississippi, Jackson, MS, USA; ^7^ Cancer Institute, University of Mississippi, Jackson, MS, USA; ^8^ Lawrence Berkeley National Laboratory, Life Sciences Division, Berkeley, CA, USA; ^9^ Buck Institute for Research on Aging, Novato, CA, USA; ^10^ Mayo Clinic, Department of Laboratory Medicine and Pathology, Division of Experimental Pathology, Rochester, MN, USA; ^11^ Mayo Clinic Cancer Center, Rochester, MN, USA

**Keywords:** heregulin, telomere, telosome, shelterin complex, TRF2

## Abstract

Telomere length, shape and function depend on a complex of six core telomere-associated proteins referred to as the telosome or shelterin complex. We here demonstrate that the isoform β2 of the heregulin family of growth factors (HRGβ2) is a novel interactor of the telosome/shelterin complex in human telomeres. Analysis of protein-protein interactions using a high-throughput yeast two-hybrid (Y2H) screen identified RAP1, the only telomere protein that is conserved from yeasts to mammals, as a novel interacting partner of HRGβ2. Deletion analysis of RAP1 revealed that the linker domain, a region previously suggested to recruit negative regulators of telomere length, interacts specifically with HRGβ2. Co-immunoprecipitation and imaging experiments demonstrated that, in addition to RAP1, HRGβ2 could associate with the RAP1-associated telomeric repeat binding factor 2 (TRF2). Deletion analysis of HRGβ2 confirmed that a putative nuclear localization signal (NLS) was necessary for nuclear HRGβ2 to exert a negative regulation of telomere length whereas the N-terminus (extracellular) amino acids of HRGβ2 were sufficient to interact with RAP1/TRF2 and promote telomere shortening. Taken together, our studies identify nuclear HRGβ2 as one of the previously unknown regulators predicted to be recruited by the RAP1 linker domain to negatively regulate telomere length in human cells. Our current findings reveal that a new, but likely not the last, unexpected visitor has arrived to the “telosome/shelterin town”.

## INTRODUCTION

Telomeres are specialized DNA-protein complexes found at the ends of eukaryotic linear chromosomes that provide protection from degradation, fusion, and recombination [1, 2]. Telomere homeostasis requires the cooperation between the DNA polymerase, telomerase, and an array of telomere end-binding factors including TRF1, TRF2, TIN2, RAP1, TPP1, and POT1. This complex, formed by multiple telomeric proteins with exquisite specificity for the arraysof telomerase-added TTAGGG repeats at chromosome ends, is known as the telosome or shelterin complex [3, 4]. Given the variety of functions that the telosome/shelterin complex performs, it is anticipated that the number of its affiliated factors will increase [3–5]. Accordingly, this complex of six core DNA-binding proteins and their sub-complexes serve as basic building blocks from which diverse signaling pathways originate through coordination of protein-protein interactions and protein complex cross-talk on the telomeres, the so-called telomere “interactome” [6–8].

The heregulin (HRG) family of growth factors comprises 15 distinct isoforms originating from alternative RNA splicingof HRG1, HRG2, HRG3, and HRG4 genes. HRG plays an important role in human cancer through its conventional paracrine or autocrine ability to bind and activate the HER-2/-3/-4 (erbB-2/-3/-4) tyrosine-kinase cell surface receptors [9–13]. Focusing on the isoform HRGβ2, our previous studies in mice have shown that HRGβ2 overexpression, independent of estrogen stimulation or high levels of the HER2 (erbB-2) oncogene, is sufficient for the generation of adenocarcinomas while favoring the metastatic spread of breast cancer(BC) cells [14, 15]. Conversely, antisense blockade of HRGβ2 expression efficiently inhibits tumorigenesis and metastasis in BC cells [16]. Therefore, HRGβ2 can exclusively promote cell malignant transformation, tumorigenicity and metastasis. Moreover, HRGβ2 can differentially modulate BC cell responses to DNA-damaging drugs. Accordingly, forced expression of HRGβ2 promotes cancer cell hypersensitization to the anthracycline antibiotic doxorubicin while inducing resistance to the alkylating agent cisplatin [17–19]. Interestingly, some HRGs have been shown to translocate to the nucleus in cancer cells, strongly suggesting that the activity HRGβ2 is not confined to the initiation of surface receptor-mediated signaling [18, 20–23]. It is not clear, however, which functions of HRGβ2 are exclusively dependent on the activation of erbB receptors, which can be solely attributed to its nuclear compartmentalization, and which HRGβ2 domains might serve to link a distinct sub-cellular localization of HRGβ2 with agiven function in cancer cells.

Using the yeast two-hybrid (Y2H) system as a high-throughput platform for interactomics, we here aimed to investigateHRGβ2 protein-interaction networks. Using the classical screen to search for pairwise interactions between HRGβ2 and putative interaction partners present in a human mammary gland cDNA library, we provide the first demonstration that HRGβ2 is a novel interactor of the telosome/shelterin complex that connects the linker domain of repressor-activator protein 1 (RAP1) [24], a region suggested to recruit negative regulators of telomere length, with the RAP1-associated factor TRF2 [24–27].

## RESULTS

### Yeast two-hybrid screening reveals an association between HRGβ2 and the telomeric protein RAP1

To identify new protein interacting partners of HRGβ2, we fused cDNA corresponding to the full-length coding sequence ofhuman HRGβ2 to the GAL4-DNA-Binding Domain (GAL4-DBD) in pGBKT7 as baitin the Y2H system to screen a human mammary gland cDNA library. From a total of 35 positive transformants, only two transformants included openreading frames (ORFs) for known genes. Strikingly, both candidates werefound to encode for telomere-related proteins. Positive clone #415 contained DNA coding for amino acid residues 116-221 at the C-terminal serine/arginine-rich domain of SC-35 (gi:33875326), a human splicing factor that has been detected in close proximity to telomeres [28]. Positive clone #480 contained a 1.2 kb insert. Amplification of this insert by 5′-RACE resulted in a cDNA fragment encoding amino acid residues 127-259 of RAP1 (gi:8102032; Fig. 1A), a negative regulator of telomere length that specifically interacts with the telomere-associated protein TRF2 [3, 24–27, 29, 30].

**Figure 1:**
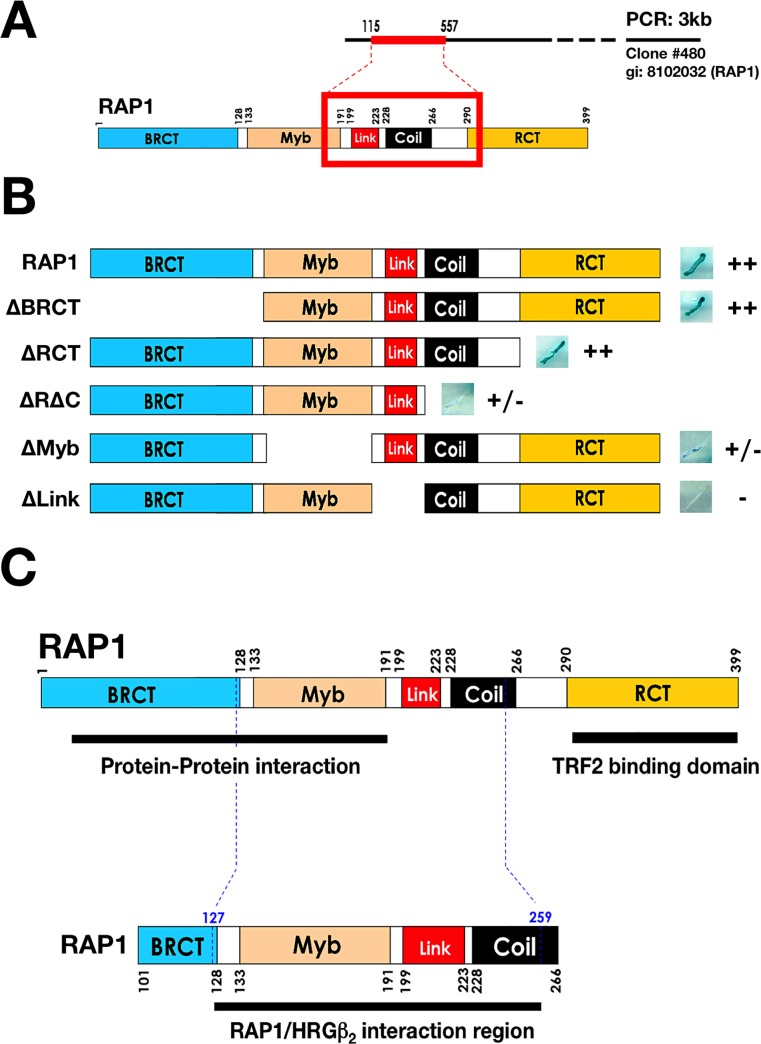
Y2H mapping of the region of RAP1 that interacts with HRGβ2 **A.** Sequencing analysis of a yeast two-hybrid screen using full-length HRGβ_2_ cDNA in the pGBKT7 vector and the human mammary gland Matchmaker cDNA library in the pACT2 vector revealed that RAP1 interacts strongly and ina domain-specific manner with HRGβ_2_. **B.** Yeast was co-transformed with plasmids encoding 1) a fusion protein between the GAL4 activation domain and full-length or deletion hRap1 constructs, and 2) a fusion protein between the pGBKT7 DNA-binding domain and full-length HRGβ_2_. Representative β-gal activity (indicated by a blue color) and growth on plates containing selective medium in the yeast two-hybrid assay are presented at the right of the scheme. ++ indicates strong interaction, +/− normal to weak interaction, and – no interaction. BRCT, Myb, Link, Coil, and RCT are RAP1 domains. **C.** RAP1 interacts with HRGβ_2_ at amino acids 127 through 259, specifically at the linker domain.

In light of these observations and because of our recent novel finding that HRGβ2 is a new regulator of telomere length, likely through its ability to regulate and interact with TRF2 and RAP1 [31], we undertook a systematic approach to better understand the role of HRGβ2 at the telosome/shelterin complex in human telomeres. To confirm the direct interaction between HRGβ2 and RAP1, we transformed yeast withthe full-length HRGβ2 ORF in the pGADT7 vector and a pACT2 plasmid expressing the full length RAP1 cDNA. Significant yeast growth was observed in plates containing selection medium (data not shown) indicating of an interaction between HRGβ2 and RAP1, and transformants were positive in the β-galactosidase assay (Fig. 1B).

### The linker domain of RAP1 mediates the interaction between RAP1 and HRGβ2

RAP1 contains four distinct functional domains: aBRCT domain, a Myb HTH motif, a coiled region, and a C-terminal proteininteraction domain (termed RCT). This structural architecture has been proposed to function as a protein adaptor, bringing different factors into the telomeric complex [24, 25, 29, 30]; for example, the RCT domain of RAP1 interacts with TRF2 and tethers RAP1 to telomeres. Moreover, deletion analysis of RAP1 has suggested that the BRCT and Myb domains, as well as a novel linker region, may modulate the recruitment of one or more protein factors, which together are required for the execution of negative telomere length control [24, 25, 29, 30]. To further characterize the interaction between RAP1 and HRGβ2, we generated deletion mutants lacking each of the four functional domains or the linker region, and used these in the Y2H system. Deletion of the RCT or the BRCT domain did not significantly affect the interaction withHRGβ2 (Fig. 1B). In contrast, deletion of either the coiled region or the Myb domain evidently reduced the interaction between RAP1 and HRGβ2. Importantly, no interaction with HRGβ2 was observed with the RAP1 construct deficient for the linker region (Fig. 1B). Therefore, while the BRCT and Myb domains of RAP1 are not sufficient tobind HRGβ2, the RAP1 linker domain appears to be essential. These results together with the sequencing data from the Y2H screen indicate that HRGβ2 might constitute a novel interactor of the telosome/shelterincomplex through its interaction with RAP1 downstream of the BCRT domain, likely between amino acids 127 and 259 (Fig. 1C).

### The N-terminal residues of HRGβ2 are sufficient to interact with RAP1 in cell nuclei

**Figure 2:**
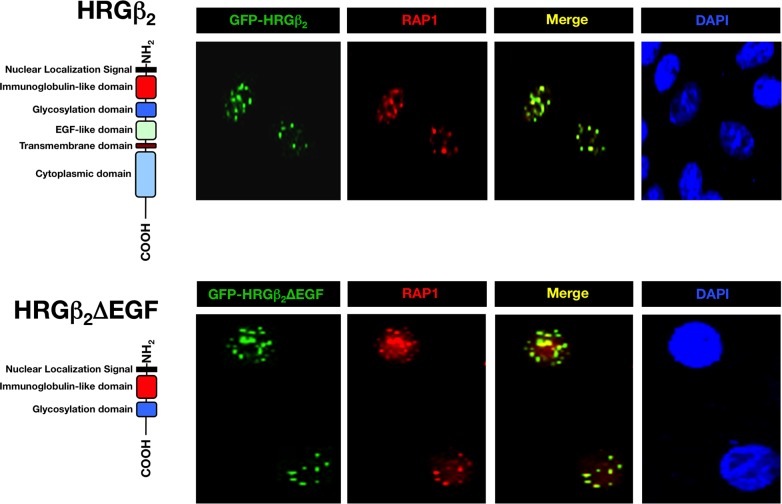
GFP-HRGβ2 and GFP-HRGβ2ΔEGF colocalize with RAP1 in MCF-7 breast cancer cells MCF-7 breast cancer cells were transiently transfected with GFP-HRGβ_2_ (*top*, green) or with GFP-HRGβ_2_ΔEGF (*bottom,* green) and were stained with an anti-RAP1 antibody (red). Colocalization of merged images is indicated in yellow*,* and DAPI staining (blue) indicates nuclear DNA. The schematic domains of HRGβ_2_ constructs are shown. Full-length HRGβ_2_ directs GFP to cytoplasmic membrane, peri-nuclear region, nucleolar-like structures and nuclear speckles. In a significant proportion of cells, the full-length form of HRGβ_2_ targets GFP to nuclear speckles, as shown in top panels. Merged images revealed co-localization (yellow) of GFP-tagged full-length HRGβ_2_ with RAP1 in many nuclear speckles. Deleting the EGF-like, the transmembrane and the cytoplasmic domains of HRGβ_2_ prevented the localization of HRGβ_2_ to cytoplasmic membrane, peri-nuclear region and nucleolar-like structures. GFP-tagged HRGβ_2_ΔEGFwas targeted to nuclear speckles only in 100% of transfected cells. Co-staining with anti-RAP1 antibody revealed that the N-terminus residues of HRGβ_2_, which are sufficient to direct the GFP tag to nuclear speckles, strongly co-localize with RAP1.

To verify the interaction between HRGβ2 and RAP1 *in vivo*, we first characterized the nuclear translocation ability of HRGβ2, which has been demonstrated for other HRG isoforms [21–23]. We transiently transfected MCF-7 BC cells with a mammalian expression plasmid encoding EGFP-tagged full-length HRGβ2 or the empty pEGFP-C2 vector and examined the distribution of EGFP by fluorescence microscopy after 24 h. In contrast to control cells, which had a uniform distribution of GFP throughout the cytoplasm (results not shown), GFP-HRGβ2-transfected MCF-7 cells showed localization of the GFP signal to distinct cellular compartments including cytoplasmic membrane, peri-nuclear region, nucleolar-like structures and nuclear speckles (Fig. 2, *top*). Importantly, immunostaining with an anti-RAP1 antibody demonstrated that GFP-HRGβ2 concentrated at nuclear speckles, mostly co-localizing with native RAP1 (Fig. 2, *top*).

To narrow down the sequence necessary for the nuclear import and sub-nuclear localization of HRGβ2, we constructed several deletion mutants of GFP-HRGβ2 (unpublished data). We found that 100% of the cells transfected with an HRGβ2-derived construct lacking the EGF-like, the transmembrane and the cytoplasmic domains (HRGβ2-ΔEGF), exhibited staining at nuclear speckles only (Fig. 2, *bottom*). Moreover, the speckled distribution pattern of GFP/HRGβ2-ΔEGF completely co-localized with endogenous RAP1 immunostaining (Fig. 2, *bottom*). Together, these results confirm that HRGβ2 appears to interact with RAP1 at cell nuclei and further reveal that the N-terminal residues of HRGβ2 are sufficient for strong co-localization with RAP1, whereas the C-terminal region of HRGβ2 is dispensable for RAP1 binding.

### HRGβ2 forms a protein complex with RAP1 *in vivo*

**Figure 3:**
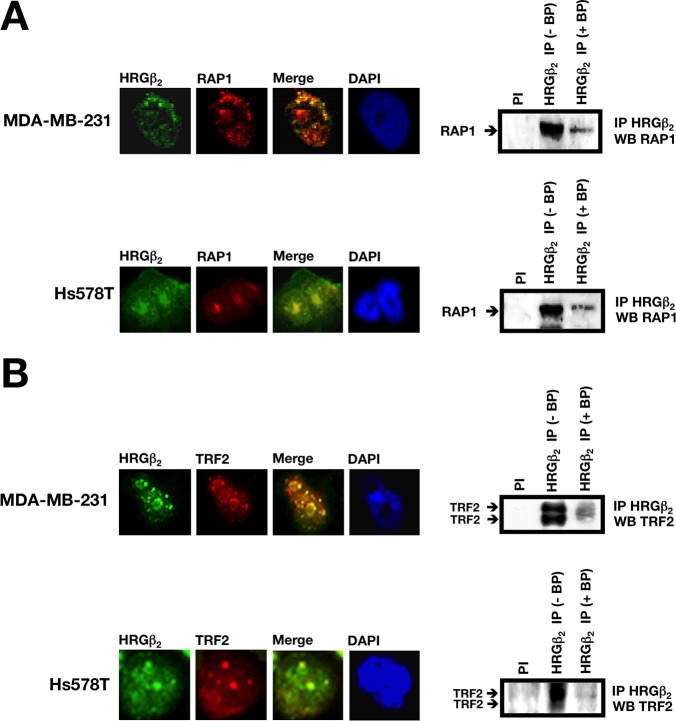
HRGβ2 co-localizes and co-immunoprecipitates with RAP1 and the RAP1-interacting partner TRF2 in breast cancer cells Representative immunofluorescent images of MDA-MB-231 and Hs578T cells co-stained for endogenous HRGβ_2_ (green) and **A.** RAP1 (red) or **B.** TRF2 (red) are shown (*n* = 3). Merged images indicate co-localization (yellow). DAPI was used to visualize nuclear DNA (blue). Endogenous HRGβ_2_ was immunoprecipitated from MDA-MB-231 or Hs578T nuclear extracts with pre-immune serum (PI) or a polyclonal anti-HRGβ_2_ antibody (HRGβ_2_ IP) in the absence (−) or presence (+) of a blocking HRGβ_2_ peptide (BP). Precipitates were separated by SDS-PAGE, transferred to nitrocellulose, and probed with antibodies against A. RAP1 or B. TRF2. One representative immunoblot is shown (*n* = 3).

To bolster our findings that HRGβ2 and RAP1 form a protein complex *in vivo*, we next performed indirect immunofluorescent studies and co-immunoprecipitation assays in MDA-MB-231 and Hs578T BC cells, two metastatic cancer cell models that naturally overexpress HRGβ2 [16]. Consistent with our results using GFP-tagged HRGβ2 protein in HRGβ2-negative MCF-7 cells, a prominent sub-nuclear localization of endogenous HRGβ2 was observed in MDA-MB-231 and Hs578T cells (Fig. 3A, *left panels*). Immunostaining with an anti-RAP1 antibody further confirmed that the punctuate distribution pattern of endogenous HRGβ2 significantly co-localized with endogenous RAP1 (Fig. 3A, *left panels*). This co-distribution of HRGβ2 and RAP1 occurred throughout the nuclei in essentially all cells, suggesting a lack of dependence on cell cycle status.

Nuclear extracts from MDA-MB-231 and Hs578T cells were immunoprecipitated with protein A/G beads coupled to an anti-HRGβ2 antibody. After elution, the purified proteins were analyzed by immunoblotting using an anti-RAP1 antibody, and compared to crude nuclear extracts or an immunoprecipitate from a non-specific antibody. Western blot analyses of proteins released from the beads revealed that RAP1 co-immunoprecipitated with HRGβ2 (Fig. 3A, *right panels*). This association was specific since no RAP1 was recovered with a pre-immune serum; moreover, HRGβ2-RAP1 co-immunoprecipitation was fully prevented in the presence of a competing peptide directed against the sequence from which the anti-RAP1 antibody was raised (Fig. 3A, *right panels*).

### HRGβ2 interacts with the RAP1 partner TRF2

Having established a physical interaction between HRGβ2 and RAP1 in the nuclei of human cancer cells, we next questioned whether HRGβ2 might also interact with the RAP1 partner TRF2. Similar to previous findings, endogenous TRF2 was found to exhibit a punctuated pattern in the nuclei of MDA-MB-231 and Hs578T cells, as demonstrated by immunofluorescent staining (Fig. 3B, *left panels*). More importantly, native TRF2 significantly co-localized with endogenous HRGβ2 (Fig. 3B, *left panels*). Furthermore, co-immunoprecipitation analysis in MDA-MB-231 and Hs578T cells specifically showed that TRF2 and HRGβ2 co-precipitate since this interaction was abolished in the presence of an HRGβ2-derived blocking peptide (Fig. 3B, *right panels*). This association between HRGβ2 and RAP1/TRF2-containing protein complexes was not due to tethering by nucleic acids since the addition of ethidium bromide to nuclear extracts during the preclearance step andthe immunoprecipitation reaction did not affect the recovery of RAP1 and TRF2 from the HRGβ2 immunoprecipitation (data not shown).

### Nuclear localization of HRGβ2 is necessary for HRGβ2-driven regulation of telomere length

**Figure 4:**
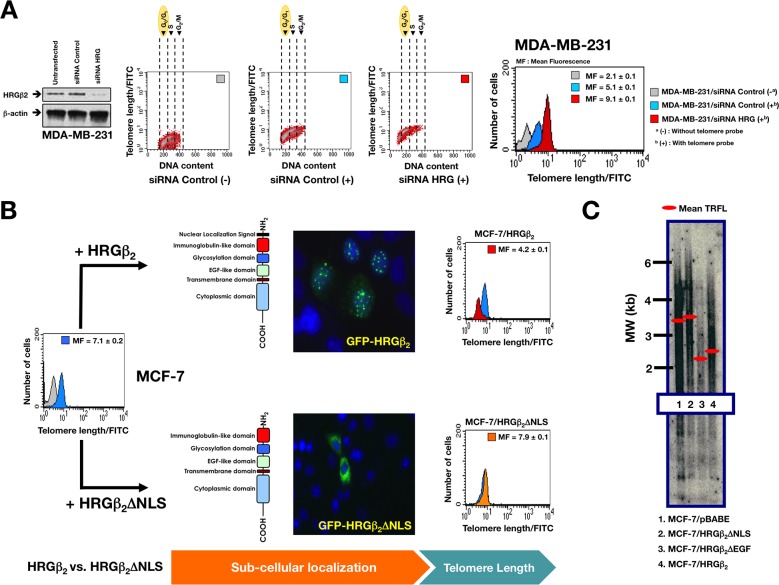
Nuclear HRGβ2 negatively regulates telomere length in breast cancer cells **A.** *Left.* Representative immunoblot showing shRNA-mediated knockdown of HRGβ_2_ in MDA-MB-231 cells. *Right.* Flow-FISH analysis of cells in the G_0_/G_1_ phase was performed using a telomere-specific probe as described in the“Materials and methods” section. (−) and (+) indicate Flow-FISH analysis in the absence or presence, respectively, of the telomeric probe. Bivariate cytograms indicate the telomere length in each sub-compartment of the cell cycle. The histograms show the telomere length within the G_0_/G_1_ cellsubpopulation, where the cells have only one copy of the genome. Telomere length is expressed in relative fluorescence units (mean ± SD) of four independent experiments. **B.** MCF-7 cells were transiently transfected with GFP-HRGβ_2_ or GFP-HRGβ_2_ΔNLS, and telomere length was analyzed by Flow-FISH as described above (n = 3). **C.** MCF-7 cells were stably transduced with either pBABE retroviral vector alone or pBABE containing cDNA for HRGβ_2_ΔNLS (a structural mutant of HRGβ_2_ that lacks the putative NLS), HRGβ_2_ΔEGF (a structural mutant of HRGβ_2_ that cannot bind HER3 or HER4 receptors), or for full-length HRGβ_2_ as specified. Telomere length was assessed by Southern blot analysis of *Hinf*I/*Rsa*I–digestedgenomic DNA as described in the “Materials and methods” section. Molecular weight (MW) markers (black bars, in kb) and mean terminal restriction fragment lengths (TRFL; red bars) are indicated.

Our discovery of the importance of the linker domain of RAP1, a region previously suggested to recruit negative regulators of telomere length [24–27], is consistent with recent findings from our group showing that the proteinaceous link between HRGβ2, TRF2, and RAP1 might explain HRGβ2's ability to negatively regulate telomere length [31]. We therefore investigated whether the nuclear co-localization of HRGβ2 with the RAP1/TRF2 complex was required for HRGβ2-driven regulation of telomere length. We first needed to confirm that HRGβ2 is a novel regulator of telomere length [31]. Using fluorescent *in situ*hybridization (FISH) with labeled peptide nucleic acid (PNA) probes specific for telomere repeats, in combination with fluorescence measurements by flow cytometry (flow FISH), we verified that transient shRNA-driven knockdown of HRGβ2 expression was sufficient to rapidly promote significant lengthening of telomeres in the highly-aggressive, HRGβ2-overexpressing MDA-MB-231 BC cell line (Fig. 4A). We then evaluated whether the minimal structural requirements for HRGβ2nuclear translocation determined its ability to regulate telomere length. We identified a putative nuclear localization signal (NLS) in the N-terminus (extracellular domain) of HRGβ2, between amino acids 4 and 16. To test the functionality of this region, we deleted the first 33 amino acids of the HRGβ2 cDNA containing this putative NLS and analysed HRGβ2 nuclear translocation. Indirect immunofluorescence of MCF-7 BC cells transiently transfected with the GFP/HRGβ2-ΔNLS fusion expression plasmid revealed that the deletion of the NLS domain completely prevented its transport to the nucleus. Accordingly, a striking peri-nuclear accumulation of GFP/HRGβ2-ΔNLS was clearly seen inmost cases in transfected cells, in comparison with the prominent nuclear speckles observed with transfected full-length HRGβ2 protein (Fig. 4B). Importantly, whereas MCF-7 cells transiently transduced to express highlevels of full-length HRGβ2 exhibited telomere shortening as revealed by a significantly lower telomeric fluorescence intensity on Flow-FISH analyses, we failed to observe significant changes in the telomere length of MCF-7 cells transiently transduced to express high levels of HRGβ2 lacking the NLS sequence (Fig. 4B). To complement this finding, we analyzed telomere length by pulse-field gel analysis of digested genomic DNA followed by Southern blot hybridizationusing a telomere-specific probe. In MCF-7 cells stably overexpressing either full length HRGβ2, HRGβ2-ΔNLS or HRGβ2-ΔEGF constructs, we found that the nuclear localization of HRGβ2 was necessary to promote telomereshortening, and the N-terminal (extracellular) amino acids of HRGβ2 aresufficient to promote this effect (Fig. 4C).

## DISCUSSION

A growing number of growth factors such as FGF, amphiregulin and PDGF have been identified in the nucleus [32–36]. The biological implications of such localizations, however, are not well understood and remain contentious. We show for the first time that HRGβ2 unexpectedly functions as an interactor of the telosome/shelterin complex in the nucleus by virtue of its direct interaction with RAP1, a negative regulator of telomere length that associates with telomeres primarily through its interaction with the telomere-binding protein TRF2. Specifically, the region of RAP1 that interacts with HRGβ2 necessarily involves its linker region. Earlier studies showed that a RAP1 mutant lacking this region was incapable of extending telomeres, and results from deletion analyses suggested that the linker region could modulate the recruitment of putative negative regulators of telomere length [24, 25, 29, 30]. Consistent with these findings, we found that changes in HRGβ2 expression and sub-cellular compartmentalization were concomitant with rapid and detectable alterations in telomere length, thus supporting thenotion that HRGβ2 acts as a previously unrecognized negative regulator of telomere length and is recruited by the linker domain of RAP1.

**Figure 5:**
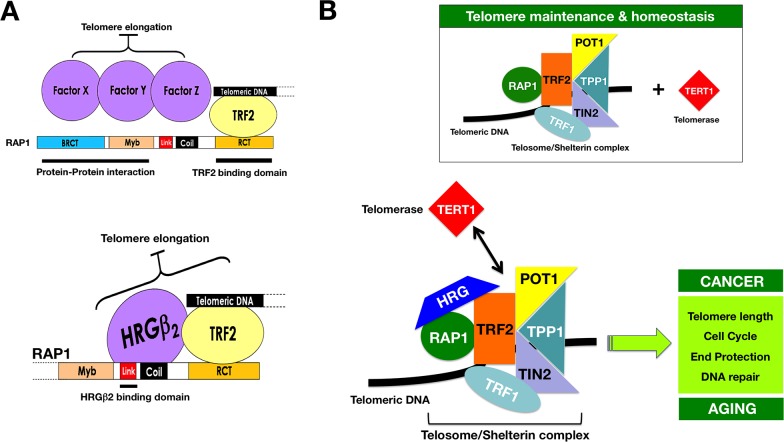
HRGβ2: A novel interactor of the telosome/shelterin complex **A.** Data reported by Li & Lange [30] and O'Connor et al. [24]originally suggested that RAP1 is a negative regulator of telomere length and that the BRCT, linker and/or Myb domains were required for this regulatory function. It was proposed that these RAP1 domains shouldinteract with a factor(s), i. e., either three different proteins, *X*, *Y*, and *Z*, or different domains of the same protein(s), which will be required fornegative regulation of telomere length. Our current mapping of the interacting regions between RAP1 and HRGβ_2_, together with the sequencing data from the Y2H screen, reveal that HRGβ_2_ isa novel interactor of the telosome/shelterin complex through its interaction downstream of the BRCT domain of RAP1, likely between amino acids 127 and 259. HRGβ_2_ should be viewed as one of the unknown negative regulators of telomere length recruited by the linker domain of RAP1 that, in addition, simultaneouslyinteracts with the RAP1 partner TRF2. **B.** The delineation of the 6 telomeric proteins (TRF1, TRF2, TIN2, RAP1, TPP1, and POT1) and their associated partners has provided the basis forconstructing an interaction map of telomere regulators in mammalian cells, the so-called telomere interactome [6–8], which represents the molecular machinery that controls mammalian telomeres and enables the integration of the various signaling pathways that regulate telomeres with other cellular interactomes. At the core ofthe telomere interactome is the telosome/shelterin complex, which can recruit a multitude of factors through various telosome subunits (e. g., RAP1) and mostly through protein hubs, which mediate multiple interactions with other proteins in the telomere interactome consistent with their key roles in telomere control (e. g., TRF2). Because TRF2 directly binds telomere DNA and recruits different proteins that are involved not only in cell cycle, DNA repair and recombination but also in end protection (e. g., RAP1) [6–8, 55], the ability of HRGβ_2_ to interact with RAP1 and TRF2 strongly suggest that HRGβ_2_ may significantly alter the protein-protein interaction within the telomere interactome to regulate telomere function and dynamics in normal development or in diseased cells, such as cancer and aging cells. Whether the RAP1-HRGβ_2_-TRF2 interaction actually determines whether the telomerase complex can be recruited or displaced from the core of the telosome/shelterin complex and/or whether TRF2 can efficiently remodel the telomere end into t-loopstructures [55], which sequester and protect the ends of chromosomes, remains to be determined.

How might HRGβ2 regulate telomere length? It hasbeen described that expression of a RAP1 mutant lacking the linker region reduced the number of TRF2 foci in cells. This result suggests that while the RAP1-Δlinker construct was still able to interact with TRF2, it may disrupt the interaction of TRF2 with the telomere and, consequently, lead to a reduction in average telomere length [29, 30]. Alternatively, the linker domain might be also involved in the recruitment of yet unidentified negative regulators of telomere length. Because our findings reveal that HRGβ2 is not a core component but rather an accessory factor of the telosome/shelterin complex, the ability of HRGβ2 to simultaneously co-interact with RAP1 and TRF2 may reconcile these two hypotheses. Suppression of HRGβ2 expression or prevention of its efficient nuclear translocation might uncouple the RAP1/TRF2 complex from sequestering the telomere ends and allow access of telomerase to its substrate. Conversely, HRGβ2 overexpression or nuclear accumulation of HRGβ2 might promote the stabilization of the RAP1/TRF2 complex at the chromosome ends, thus leading to telomere shortening. In line with this concept, we found that forced expression of HRGβ2 in HRGβ2-negative BC epithelial cells is sufficient to induce the up-regulation and the stabilization of RAP1/TRF2 complexes while coincidentally promoting telomere shortening. Knockdown of HRGβ2, however, decreases the abundance of TRF2 and RAP1 proteins and reduces their telomeric content while concomitantly inducing telomere lengthening [31]. These RAP1/TRF2-related telomere-regulating actions of HRGβ2 recapitulate the features found by deleting the linker region of RAP1 [24]. Since most if not all TRF2 is in a complex with RAP1, it is reasonable to suggest that HRGβ2 might function as a direct interacting partner forstable binding between RAP1 and TRF2. In this scenario, HRGβ2 could function to modulate correct telomere tethering of this telosome/shelterin complex subunit and, ultimately, regulate telomere homeostasis maintenance (Fig. 5A).

Our current findings revealing the telomere-related “intracrine” functioning of HRGβ2 supports the concept that, in a counterintuitive manner, secreted growth factors such as the HRG family, can promote highly specific actions in defined cellular compartments. This discovery adds even more complexity to the “import(ance) of growth factors in(to) the nucleus” [32–36]and may have significant implications in our molecular understanding ofthe natural history of age-related pathologies including cancer. Indeed, our current findings provide molecular support to our “telomere-dependent” hypothesis, integrating epithelial BC, aging stromaand cellular senescence, by considering an unforeseen telosomic activity of HRG [37–40]. Earlier studies from our laboratory described how senescent human fibroblasts significantly stimulated hyperproliferation and progression of pre-neoplastic breast epithelial lesions and accelerated the tumorigenic capacity of neoplastic breast epithelial cells [41]. The BC-promoting activity of senescent cells was likely due to the secretory phenotype of senescent fibroblasts, which is characterized by changes in the expression of cell type-specific genes [39, 42, 43]. While acknowledging that the critical factor(s) produced by aging–senescent- stroma for the stimulation of pre-cancerous and cancerous breast epithelial lesions remains an unanswered question, it is relevantto note that HRG is one of the major growth factors secreted by senescent fibroblasts [42, 44]. Beyond the well-characterized autocrine/paracrine activating actions ofHRG on the erbB network-driven signaling, its intracrine regulation of telomere-dependent genome stability may provide a key rate-limiting stepin breast carcinogenesis and metastatic progression. Short telomeres have been shown to generate chromosome instability, which in turn may lead to an increased risk of several cancers including breast carcinoma [45–47]. As an illustration, we now know that transition through telomere crisisand immortalization is a crucial event in BC that occurs during progression from typical ductal hyperplasia to pre-invasive ductal carcinoma *in situ* (DCIS), as well as in focal areas of histologically-normal epithelium from which breast carcinoma is thought to arise [48, 49]. In this scenario, an HRG-induced shortening of telomeres in breast epithelial cells, as an additional driver of chromosome destabilization in the early stages of breast tumorigenesis, may molecularly link the tumorigenic role of HRG-overproducing senescent cells at sites of age-related pathologies such as BC. Indeed, a reduced telomeric DNA content correlates with genomic instability and metastasis in invasive breast carcinomas [50, 51]. Remarkably, pro-metastatic HRG proteins have recently been found to significantly accumulate not only in cell nuclei of invasive breast carcinomas but also in DCIS, whereas no nuclear HRG is found in normal tissues [22, 52]. Therefore, it is reasonable to suggest that some cancer promoting actions of the aging stroma can rely, at least in part, on its ability to secrete enormous amounts of HRG which, in turn, will trigger and maintain telomere shortening in the breast epithelial compartment in a RAP1/TRF2-dependent manner.

The elucidation of the interaction and function of the core proteins TRF1, TRF2, RAP1, TIN2, POT1, and TPP1 within the telosome/shelterin complex has allowed a better understanding of the molecular intricacies that ultimately regulate telomere end-capping and length control. It is now known that the telosome/shelterin complex forms the basic platform on which layers of telomere signaling networks can be assembled onto the so-called telomere interactome [6, 7]for the proper protection and maintenance of mammalian telomeres. Considering the ever-growing list of proteins that integrate the complexand labyrinthine network of the telomere interactome, one can wonder “how there is even room” for new regulators at chromosome ends [53, 54] (Fig. 5B). Our study refines the current appreciation of the telomere interactome by incorporating the nuclear version of the growth factor HRG as a constituent of the various telomere-signaling pathways and telomere-associated molecular machinery that regulate mammalian telomeres. Future studies are warranted to explore in detail the function of HRG at telomeres and to dissect the mechanisms by which HRG communicates with the telosome/shelterin complex core to coordinate different cellular signaling pathways for telomere maintenance includingcell cycle, telomere length, end protection or DNA repair in cancer andaging. Our current findings reveal that a new, but likely not the last, unforeseen player has been added to the “telosome/shelterin town”.

## MATERIALS AND METHODS

### Cloning of HRGβ2

Poly (A) RNA was extracted from HRGβ_2_-overexpressingMDA-MB-231 BC cells using the Oligotex Direct mRNA Kit (Qiagen). Three μg of poly (A) RNA was reverse transcribed and amplified using the Gene Amp RNA PCR Kit (Applied Biosystems). To obtain the entire ORF of HRGβ_2_, the digested PCR product was cloned into the pRcCMV plasmid (Met1 to Ser422) using the BSU361 and *Xba*I sites. The region encoding the C-terminal domain of HRGβ_2_ (Phe401 to Val636) was amplified using primers 5′- TCAGGCATGCCAGAGAAACC-3′ and 5′-ATTATACAGCAATAGGGTCTTGGTT-3′.

### Yeast two-hybrid assay

The yeast two-hybrid assay was performed according to the Matchmaker Gal4 two-hybrid system 3 manual (CLONTECHniques). The yeast strain AH109 containing *ADE2*, *HIS3*, *lacZ*, and *MEL1* reportergenes, each of which uses a distinct GAL4-responsive promoter, was usedin the assay. These distinct promoter elements automatically eliminate three major categories of false positives: those caused by proteins thatinteract upstream of the reporter construct binding site, those that interact directly with the sequences flanking the GAL4 binding site, andthose that interact with transcription factors bound to specific TATA boxes. The entire HRGβ_2_ ORF was sub-cloned in frame into the DNA-Binding Domain (BD) vector pGBKT7. The pGBKT7-HRGβ_2_ bait vector and the human mammary gland Matchmaker cDNA library in the pACT2 vector were co-transformed into strain AH109 and grown on minimal synthetic medium lacking Ade, His, Leu and Trp to test potential interactors. The HRGβ_2_ bait did not have any intrinsic activity of transcriptional activation for the reporters (data not shown). The X-α-Gal (5-Bromo-4-chloro-3-indolyl-α-D-galactopyranoside) indicator was included to screen transformants.

Approximately 1 × 10^6^ independent transformants were pooled and re-plated on selection media (Ade-, His^−^, Leu^−^ and Trp^−^) containing 2% galactose (Gal) to induce the expression of cDNAs. In total, 35 transformants showed Gal-dependence in the selction medium. Plasmids from these transformants were extracted by yeast mini-prep and the cDNAs were amplified in the PCR with primers derived from the pJG4-5vector, followed by sequence determination. To eliminate false positive, we performed separation and isolation of yeast plasmids followed by propagation in bacteria. This approach revealed that: 1 clone was not in frame with GAL4, 6 clones included 3′ untranslated regions (UTRs), while 24 clones were found to be artifactual positive yeast clones. The two truly positive clones including ORFs for known genes were found to encode for the telomere-related proteins SC-35 and RAP1 (see text).

Mapping of the interacting domains of RAP1 and HRGβ2. AH109 cells were co-transformed with plasmids encoding a fusion protein between the GAL4 activation domain (AD) and full-length RAP1 (pACT2-hRap1, a gift from Dr Titia de Lange), or RAP1 deleted constructsand a plasmid encoding a fusion protein between full-length HRGβ_2_ and the DNA-BD included in pGBKT7, using a small-scale LiAc/ssDNA/PEG transformation protocol. RAP1 truncations were generated by PCR as described (24): hRap1 (full-length, amino acids 1-198), ΔMyb (amino acids 1-132 and 192-399), ΔLink (amino acids 1-198 and 224-399), ΔRCT (amino acids 1-290, with an N-terminal nuclear localization signal PRRK), ΔRΔC (ΔRCT/ΔCoil-Coil, amino acids 1-227, with an N-terminal nuclear localization signal PRRK), and ΔBRCT (amino acids 129-399). Transformants were plated onto appropriate minimum synthetic dropout media and then tested for β-galactosidase activity.

### Generation and visualization of GFP-HRGβ2

A full length HRGβ_2_ enhanced green fluorescent protein (EGFP) fusion construct was engineered by removing the HRGβ_2_ insert from pGBKT7/HRGβ_2_ with *Eco*RI and *Bam*HI and cloning into the equivalent sites of pEGFP-C2. Structural deletion mutants of HRGβ_2_ were generated by PCR and cloned into pEGFP-C2 or pEGFP-N2 vectors digested with *Eco*RI and *Bam*HI.

MCF-7 cells were used for transient expression analysis of GFP-HRGβ_2_ constructs. Cells were grown on glass coverslips to 60%-80% confluence and were transfected for 6 hours with 1 μg plasmid DNA using FuGENE transfection reagent (Roche, Indianapolis, IN). Transfected cells were seeded on glass coverslips in six-well plates and incubated for 24 hours. Cells were fixed/permeabilized with 0.2% Triton X-100/2% paraformaldehyde and permeabilized again in 100% methanol for 10 minutesat −20°C. Coverslips were blocked for 30 minutes at 37°Cin 5% BSA in phosphate-buffered saline (PBS), incubated with a RAP1-specific antibody (1:200 dilution of IMG-272, Imgenex, San Diego, CA), washed, and incubated with Texas Red-conjugated anti-mouse IgG. Coverslips were mounted with 4,6-diamidino-2-phenylindole (DAPI) onto microscope slides. Cells were observed and analyzed using a Zeiss Axioskop microscope equipped for epifluorescence.

### Fluorescence microscopy

MDA-MB-231 and Hs578T cells were grown on glass coverslips to 60%-80% confluence and fixed/permeabilized/blocked as described above. Cells were then stained with HRGβ_2_-, RAP1- and/or TRF2-specific antibodies (1:100 dilution of the anti-HRGβ_2_C20 antibody –sc-348-; Santa Cruz Biotech. Santa Cruz, CA and 1:200 of anti-RAP1 or anti-TRF2 IMG-124 antibody, Imgenex, San Diego, CA) and then incubated with Texas Red-conjugated anti-mouse IgG for hRap1 and TRF2 (*red*) and FITC-conjugated anti-rabbit IgG antibody for HRGβ_2_ (*green*).

### Immunoprecipitation and western blotting

For co-immunoprecipitation (IP) analysis of HRGβ_2_ and RAP1/TRF2, MDA-MB-231 and Hs578T cultures were expanded on 15-cm plates. At confluence, cells were trypsinized, collected in a10X pellet volume of PBS and lysed using the EZ Prep Nuclei Isolation Kit (Sigma-Chemicals, St. Louis, MO). The purified nuclei were resuspended in a 3X pellet volume in low salt nuclear extraction buffer [20 mmol/L Tris, pH 7.3, 20 mmol/L KCl, 25% glycerol, 1.5 mmol/L MgCl_2_,0.2 mmol/L EDTA, 1X phenylmethylsufonyl fluoride, and a complete protease inhibitor mixture tablet]. An equal volume of high salt nuclearextraction buffer (1.2 M KCl, 20 mmol/L Tris, pH 7.3, 25% glycerol, 1.5mmol/L MgCl_2_, 0.2 mmol/L EDTA) was added and the nuclear extract was kept on ice for 30 min with occasional vortexing. The sample was then centrifuged at 14,000 rpm for 15 minutes and the supernatant was diluted with an equal volume of water. One mg ofnuclear protein was pre-cleared using 1.25 μg of rabbit IgG and 20 μL of A/G beads for 30 min at 4°C (pre-immune –PI-). The supernatant was incubated for 2 hours with 1 μg of C-20 anti-HRGβ_2_ antibody (sc-348; Santa Cruz Biotech.) in the absence or presence of an HRGβ_2_ blocking peptide. Twenty μL of A/G beads were added and the mixture was incubated overnight at 4°C. Ethidium bromide or RNase A was added to the nuclear extracts (both at afinal concentration of 100 μg/ml) during preclearance and throughout the IP procedure. Immunocomplexes were washed several times with NETN buffer [20 mmol/L Tris, pH 8.0, 100 mmol/L NaCl, 0.5% Nonidet P-40, 1 mmol/L EDTA), boiled in SDS loading buffer, separated on 10% SDS-PAGE gels and transferred to nitrocellulose membranes. Nonspecific binding was minimized by blocking the membranes for 1 hr at room temperature (RT) with TBS-T [25 mmol/L Tris-HCl, 150 mmol/L NaCl, pH 7.5, and 0.05% Tween 20] containing 5% (*w/v*) nonfat dry milk. The treated filters were washed in TBS-T and then incubated with anti-hRap1 and anti-TRF2 primary antibodies for 2 hr at RT in TBS-Tcontaining 1% (*w/v*) nonfat dry milk. After a subsequent wash in TBS-T, horseradish peroxidase-conjugated secondary antibodies (Jackson Immunoresearch Labs, West Grove, PA) in TBS-T were added for 1 hr, and immunoreactive bands were detected by enhanced chemiluminescence reagent (Pierce, Rockford, IL).

### Generation of MCF-7 cells overexpressing full-length HRGβ2 or HRGβ2 deletion mutants

The PCR products generated using the HRGβ_2_ cDNA accession number 183996 (full length HRGβ_2_) or the structural deletion mutants (HRGβ_2_-ΔNLS and HRGβ_2_-ΔEGF) were cloned into the retroviral expression vector pBABE-Puro using *Bam*HI and *Eco*RIrestriction sites. Retroviral constructs were transfected into a high efficiency transient amphotropic packaging system (TSA54 cell line) withFuGENE reagent. Retrovirus-containing medium collected after 48 h was used to infect MCF-7 cells for 24 h in the presence of Polybrene (Sigma-Chemicals). Infected MCF-7 cells were grown for an additional 24 hours in standard medium and stable cell lines (MCF-7/pBABE, MCF-7/HRGβ_2_, MCF-7/HRGβ_2_-ΔNLS and MCF-7/HRGβ_2_-ΔEGF) were selected and expanded in the presence of 2.5 μg/mL puromycin for two weeks. Expression levels of HRGβ_2_, HRGβ_2_-ΔNLS and HRGβ_2_-ΔEGFwere assessed by RT-PCR using the Gene Amp Kit (Promega Corporation). Alternatively, cells were transiently transduced with replication-incompetent amphotropic retroviruses containing either a full-length HRGβ_2_ cDNA construct or a HRGβ_2_-ΔNLSdeletion cDNA construct cloned in the pLXSN (LTR-gene X-simian virus 40-Neo) retroviral expression vector (Clontech) in the presence of 4 μg/mL polybrene. Viruses were added at appropriate dilutions, and the cells incubated for 48 h to allow expression of the HRGβ_2_ or HRGβ_2_-ΔNLS proteins from the integrated pLXSN vector.

### HRG gene silencing by small interfering RNAs

An siRNA duplex used for silencing the human HRGwas purchased from Santa Cruz Biotech. Transfections were performed as described in the technical bulletin.

### Telomere analysis by fluorescence in situ hybridization and flow cytometry

Analysis of telomeres by flow cytometry using FISH was performed with a kit from DakoCytomation Denmark A/S (Glostrup, Denmark), Code No. K5327. Briefly, ∼ 2 × 10^6^ cells were resuspended in 300 μL of hybridization buffer (70% deionizedformamide, 10 mmol/L Tris, pH 7.0, 10% fetal bovine serum, FBS) in the absence or presence of 0.3 μg/mL of the telomere-specific fluorescein isothiocyanate-conjugated probe (fluorescein isothiocyanate-O-CCCATAACTAAACAC-NH_2_). DNA from samples was heat-denatured for 10 minutes at 82°C, followed by hybridization for 2 hours at RT. Cells were washed in wash buffer containing 70% deionized formamide, 10 mmol/L Tris, pH 7.0, 10% FBS and 0.1% Tween 20. After incubation for 1 hour at RT, cells were washed and resuspended in PBS, 10 % FBS, 10 μg/mL RNase A (Roche Molecular Diagnostics), and propidium iodide, incubated for 1 hour at RT, washed, and analyzed in a FACScalibur flow cytometer (Becton Dickinson). Cell Quest software (Becton Dickinson) was used for data acquisition and analysis. The telomere fluorescence signal was defined as the mean fluorescence signal in G_0_/G_1_ cellsafter subtraction of the background fluorescence signal (FISH procedurewithout probe). The flow cytometer was calibrated every day using fluorescein isothiocyanate-labeled fluorescence Sphero microparticles (Pharmingen). The resulting calibration curve was used for correction ofexperimental fluorescence values in each experiment. Green fluorescencewas measured in a linear scale, and results were expressed in molecularequivalents of soluble fluorochrome units (kMESF).

### Southern blotting analysis of Terminal Restriction Fragment (TRF) length

Cells from a sub-confluent 100-mm diameter culture dish were harvested by trypsinization, washed in cold PBS, collected by centrifugation for 5 min at 1,500 rpm, and counted using the Coulter counter. Cell pellets were frozen at −80°C until utilization. Genomic DNA was isolated from cell pellets (approximately 5 × 10^6^ cells for each cell line) using a DNA extraction kit (Qiagen, Santa Clarita, CA) with inclusion of an RNase A digestion step, and quantitated with a spectrophotometer. Protein-free DNA was cleaved with *Hinf*I and *Rsa*Irestriction enzymes (Gibco/BRL), extracted once with phenol/chloroform/isoamyl alcohol, ethanol precipitated, and resuspendedin Tris-EDTA to generate the TRF. A portion of DNA was subjected to electrophoresis in a 0.5% agarose gel in 1X Tris-Borate EDTA (TBE) buffer at 2 V cm^−1^ for 17 hours. The gel was dried at 60°Cfor 2 hours, denatured for 30-60 minutes in 0.5 mol/L NaOH and 1.5 mol/L NaCl, and neutralized for 30-60 minutes in 1 mol/L Tris-HCl, pH 8.0 and 1.5 mol/L NaCl. The dried gel was then subjected to in-gel hybridization with a [γ-^32^P]-ATP 5′ end-labeled oligonucleotide telomeric probe [γ-^32^P-(TTAGGG)_3_]. The autoradiography signal was digitized using a Phosphoimager (Amersham Biosciences) and Image-QuaNT software (Molecular Dynamics). The mean size of the TRF was estimated using the formula L = E(OD_i_ •L_i_)/ E(OD_i_), where OD_i_ and L_i_ werethe signal intensity and TRF length, respectively, at position i on thegel image. This method takes into account the greater intensity of signals from larger fragments. Briefly, the scanned image was divided into a grid consisting of *X* columns and multiple rows where *X*were the number of samples. The rows were positioned to cover the entire vertical length of the image. The vertical size of grid boxes wasarbitrary but it was small enough such that many boxes overlaid a signal smear. Two rows above and two rows below each signal smear were used to calculate the background. The background OD was averaged and then subtracted from the OD of each grid box to give the signal due to TRFs at that position. For each sample, OD and L were computed for each grid box, where OD was the total signal intensity within a grid box and Lwas the distance (in cm) at the mid-point of the grid box. Mean TRF length was then calculated using the above formula and values reported are the average of at least two independent Southern blots.
